# Adjuvant antimicrobial photodynamic therapy improves periodontal health and reduces inflammatory cytokines in patients with type 1 diabetes mellitus^*^

**DOI:** 10.1590/1678-7757-2024-0258

**Published:** 2024-10-04

**Authors:** Paula de Oliveira CUNHA, Isabela Rodrigues GONSALES, Sebastião Luiz Aguiar GREGHI, Adriana Campos Passanezi SANT’ANA, Heitor Marques HONÓRIO, Carlos Antonio NEGRATO, Mariana Schutzer Ragghianti ZANGRANDO, Carla Andreotti DAMANTE

**Affiliations:** 1 Universidade de São Paulo Faculdade de Odontologia de Bauru Departamento de Prótese e Periodontia Bauru Brasil Universidade de São Paulo, Faculdade de Odontologia de Bauru, Departamento de Prótese e Periodontia, Bauru, Brasil.; 2 Universidade de São Paulo Faculdade de Medicina de Bauru Bauru Brasil Universidade de São Paulo, Faculdade de Medicina de Bauru, Bauru, Brasil.

**Keywords:** Photodynamic Therapy, Lasers, Periodontitis, Diabetes Mellitus, Type 1, Cytokines

## Abstract

Antimicrobial photodynamic therapy (aPDT) is an adjuvant treatment to scaling and root planing (SRP) which improves periodontal health. It may be beneficial to patients with systemic diseases, such as type 1 diabetes mellitus.

**Objective:**

This randomized clinical trial evaluated the adjunctive effect of aPDT on the periodontal treatment of patients with type 1 diabetes (T1D).

**Methodology:**

38 patients were included in the study and divided into four groups: DSRP – T1D patients treated with SRP; CSRP – normoglycemic patients treated with SRP; DPDT – T1D patients treated with SRP + aPDT (methylene blue and red laser); CPDT – normoglycemic patients treated with SRP + aPDT. , Periodontal clinical parameters and inflammatory cytokines in crevicular fluid were recorded at baseline and then after 1, 3 and 6 months. The clinical endpoint for treatment was evaluated after 6 months.

**Results:**

Adjuvant aPDT treatment resulted in reduction of probing depth after 3 months (0.38 mm - p<0.05) on T1D patients and in control group after 6 months (0.66 mm - p<0.05). Reduction of clinical attachment levels was similar for both treatments in control patients (p>0.05). There was a significant reduction of TNF-α in crevicular fluid in both groups treated with aPDT (p<0.05). The T1D (65%) and normoglycemic (72%) groups achieved the clinical endpoint after both treatments (p>0.05).

**Conclusions:**

Adjuvant aPDT provided additional benefits in improving periodontal clinical parameters and reducing inflammatory cytokines in both T1D and normoglycemic patients. However, normoglycemic patients showed greater clinical improvements compared to T1D patients following adjuvant aPDT treatment.

## Introduction

Periodontitis is a comorbidity associated with diabetes, and it tends to be more severe in individuals with this condition.^1^ It is characterized by a dysbiotic microbiome and a chronic inflammatory response by the host, leading to the destruction of surrounding tooth tissues including periodontal ligament and alveolar bone. Diabetes is characterized by either the absence or impaired tolerance to insulin, which can trigger a systemic inflammatory and immune system impaired response. These systemic inflammatory processes can affect distant organs, and as such the inflammatory burden has impact on both periodontitis and diabetes.^2^ Patients with diabetes and poor glycemic control have a higher quantity of periodontopathogens, indicating that diabetes also affects the periodontal microbiota.^2^ Poorly controlled diabetes exacerbates periodontitis through the elevated production of inflammatory cytokines and high levels of advanced glycation end products in periodontal tissues. These end products trigger an exaggerated inflammatory state and oxidative stress, which affect fibroblast and other cells, ultimately leading to increased periodontal destruction.^2^

In the new classification of periodontitis, diabetes is considered a grade modifier (incorporation of individual patient factor into the diagnosis), with HbA1c <7% classified as grade B and HbA1c > 7% as grade C, and it is associated with moderate to rapid rate of periodontitis progression.^3^ Patients with type 1 diabetes (T1D) are more than twice as likely to be affected by periodontitis compared to non-diabetic patients.^4^ These patients may experience greater tooth loss due to diabetes and periodontitis onset in younger ages compared to patients with type 2 diabetes (T2D).^5^ Therefore, patients with a history of T1D diagnosis for more than 10 years experience greater loss of periodontal support.^5^

As inflammation plays a key role in periodontitis and diabetes, periodontal treatments that reduce the inflammatory burden should be the first choice for patients with diabetes. This is particularly important for patients with T1D, as one in five patients is also affected by periodontitis.^4^

Antimicrobial photodynamic therapy (aPDT) is an adjuvant treatment for periodontitis in combination with scaling and root planing (SRP). This treatment combines a photosensitizer with laser to produce a photochemical reaction that releases reactive species of oxygen and singlet oxygen, all of them toxic for microorganisms.^6,7^ Additional positive effects of this therapy on treatment of periodontitis were confirmed by clinical studies,^8-12^ and it has been found to promote inflammation control during the periodontal maintenance by reduction of inflammatory cytokines in normoglycemic patients.^13^

To the best of our knowledge, few reports in literature discuss adjuvant aPDT for treatment of periodontitis in patients with T1D, which is not the case for studies on patients with type 2 diabetes. One study demonstrated improved periodontal clinical parameters in patients with type 1 diabetes treated with adjuvant aPDT.^12^ Due to the limited investigation on this topic, it is necessary to extrapolate results from studies involving patients with type 2 diabetes. The additional effects of aPDT as an adjuvant to SRP for treating periodontitis in patients with type 2 diabetes remain controversial . Some studies did not find positive results of this association either in periodontal clinical parameters^14-16^ or in metabolic^14,15^ and microbiologic analysis.^16^ Conversely, aPDT promoted a reduction in periodontal clinical parameters in uncontrolled glycemia^17,18^ as well as reduction in *P. gingivalis* and *T. forsythia,*^17^ and in the total count of periodontopathogenic bacteria.^19^ Systematic reviews have not found additional benefits of adjuvant aPDT in the treatment of periodontitis in patients with T2D. Despite their conclusion, the authors stated that the scientific evidence is lacking, with only four studies included and short follow-up period. They also suggested that aPDT could be an important therapy for controlling periodontitis in patients with diabetes.^20^ In contrast to those findings, a recent meta-analysis confirmed that aPDT contributes to the improvement of periodontal health in T2D patients.^21^ If aPDT has additional positive effects on the treatment of periodontitis in patients with type 2 diabetes, studies involving T1D patients with type 1 diabetes are encouraged, as this population tends to have worse periodontal health and greater loss of teeth than T1D patients. It is also necessary to construct new evidence of non-surgical periodontal treatment on patients with type 1 diabetes mellitus with adjuvant treatment of aPDT.

To address this gap in the literature, this study seeks to compare the effects of adjuvant aPDT on treatment of periodontitis in patients with type 1 diabetes compared to normoglycemic patients.

## Methodology

This is a study designed as four-armed parallel randomized clinical trial. This study was approved on Ethical Committee on Human Research at the Bauru School of Dentistry (Protocol # 1.959.932 - 03-10-2017). The study follows the standards of Declaration of Helsinki. All participants gave their informed consent prior to their inclusion in the study. It is registered on Clinical trials.gov as NCT03102892. All patients were examined and treated at the Periodontology graduation clinic at the Bauru School of Dentistry from 2017 to 2021 (interruptions occurred due to the COVID-19 pandemic).

The inclusion criteria for the test group were a diagnosis of T1D signed by an endocrinologist, age between 18 and 70 years, presence of at least four teeth (one in each hemi-arch), and the presence of stage III or IV periodontitis. The exclusion criteria for the test group included a diagnosis of T2D, edentulous patients, smokers, pregnancy, other endocrine and systemic blood diseases, and the consumption of drugs that alter the metabolism of periodontium. For the control group, the inclusion criteria were absence of systemic diseases, while the other inclusion and exclusion criteria were the same.

Patients were allocated in four groups:

DSRP – T1D patients and SRP treatment.

DPDT - T1D patients and SRP + aPDT

CSRP – control normoglycemic patients and SRP treatment.

CPDT - control normoglycemic patients and SRP + aPDT

Evaluation and treatment took place over seven sessions. During the 1^st^ session, anamnesis and clinical examination were conducted. A calibrated examiner recorded the following periodontal clinical parameters at six sites/tooth, excluding 3^rd^ molars and dental implants, using a periodontal millimeter probe: probing depth (PD), clinical attachment level (CAL), bleeding on probing (BOP), plaque index (PI) (this parameter considers only four sites/tooth). During the 2^nd^ session, treatments were initiated and then repeated after seven days (3^rd^ session) and then at 14 days (4^th^ session).^20^ Reevaluation (5^th^ session) took place one month after the completion of active periodontal therapy. The follow-up sessions, including registration of periodontal parameters and prophylaxis, were conducted after 3 months (6^th^session) and 6 months (7^th^ session). The examiner’s calibration consisted in two measurements of periodontal parameters at the same patient within 15 days (Kappa value = 0.9).

Treatments consisted of SRP with Gracey curettes (Hu-Friedy, Frankfurt, Germany) and an ultrasound device (Dabi Atlante, Ribeirão Preto, Brazil) in all periodontal sites. For the aPDT group, SRP was followed by irrigation of all periodontal sites with a solution of methylene blue (10mg/ml – Sigma Aldrich – in deionized water).^10^ The pre-irradiation time was 1 minute. Afterwards, a red diode laser (InGaAlP) (Therapy XT- DMC – São Carlos/SP – Brazil) was applied using an optical fiber.^23^ Apico-coronal sweeping movements were performed from the bottom to the top of the periodontal pocket for 80 s per tooth (40 s at buccal site, 40 s at lingual site). Laser parameters were as follows: wavelength 650 ± 10 nm, power 100 mW, internal fiber tip diameter 600 µm, optic fiber area 0.028 cm^2^, total energy 8 J, energy density 285.7 J/cm^2^, power density 3.57 W/cm^2^. SRP and SRP + aPDT sessions were repeated twice after the initial treatment (for a total of three sessions, with one session every seven days),^22^ but only in periodontal pockets with a PD ≥4mm.

Collection of gingival crevicular fluid (GCF) was performed in three sites/patient. The deepest site (measured by probing depth) was selected from three different quadrants. After cotton rolls were applied to prevent contact with saliva, a paper cone was inserted into the periodontal pocket for 30 s. Samples were stored in a solution of PBS and protease inhibitor at −80 °C. After lyophilization of the samples, the inflammatory cytokines IL-1β, IL-4, IL-6, IL-8 and TNF-α (Milliplex MAP, USA) were quantified using a multiple analyte profiling Luminex assay.

Primary outcomes included a reduction in PD and CAL, while secondary outcomes were the reduction in BOP and PI, cytokine quantification and the achievement of clinical endpoint for treatment (≤4 sites with PD 5mm) after 6 months.^23^

The sample size was calculated, considering relevant a clinically relevant difference of 1 mm between groups and a standard deviation of 0.84 based in a previous study.^24^ With a power of 80% and a significance level of 5%, 52 patients (13 patients per experimental group) were required.

Computer-generated random numbers were utilized for simple randomization of participants. Efforts were made to ensure uniformity in the treatment groups (SRP or aPDT) by matching them for similar mean probing depths (p>0.05) at baseline. An individual not affiliated with the research group generated the random allocation, which was concealed in an envelope until the time of treatment. In addition, a block randomization was conducted according to the severity of periodontal disease. An equal number of patients presenting stage III or IV periodontitis were allocated to each treatment group (aPDT or control).

This is a double-blinded study. Distinct researchers were responsible for administering interventions, collecting data and conducting statistical analysis. Patients were not blinded as the therapies were substantially distinct. Antimicrobial photodynamic therapy involves irrigation with a blue photosensitizer that temporarily stains the oral mucosa and imparts a bitter taste. Additionally, the application of laser requires the use of safety glasses.

Statistical analysis was conducted with the experimental unit defined as the number of patients (n=38) and the number of sites (n=5,441).

- T1D patients (n=18): DSRP - 8 patients and 1,122 sites. DPDT - 10 patients and 1,524 sites.
- Control patients (n=20): CSRP - 10 patients and 1,409 sites. CPDT - 10 patients and 1,386 sites.

All analyses were conducted using repeated measures ANOVA complemented by Tukey’s post hoc test (p<0.05). Cytokine quantification was analyzed by repeated measures ANOVA complemented by Fisher’s post hoc test (p<0.05). The achievement of clinical endpoint for treatment was assessed by chi-square and Fisher’s p-value tests (p<0.05). Potential correlations between PD and cytokine levels on each site (three sites/patient) were examined using Fisher’s correlation test (p<0.05) (Statistica 10.0). Different statistical approaches were used to reduce the risk of bias.

## Results

**Figure 1:**
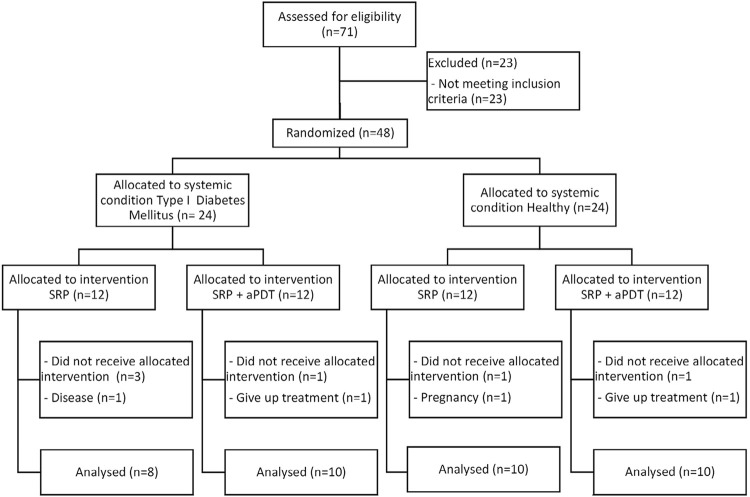
Consort flow diagram of the study showing randomization, allocation and interventions. (SRP – scaling and root planing; aPDT – antimicrobial photodynamic therapy)

A total of 38 patients were enrolled in the study, comprising 20 normoglycemic individuals (8 male and 12 female, mean age 42.1 years) and 18 individuals (8 male and 10 female, mean age 40.8 years) with T1D. The mean duration since the diagnosis of diabetes was 17.63±11.71 years for the SRP group and 24.5±11.01 years for the aPDT group. Figure 1 illustrates the patient flow diagram. For the DSRP group, the mean HbA1c was 9.2±1.35%, while for the DPDT group, it was 8.7±1.75% (which indicates uncontrolled diabetes). All patients with diabetes used insulin.

### Clinical parameters

Table 1 illustrates a significant reduction in mean PD after treatment up to 6 months (p<0.05). The aPDT groups exhibited greater PD means at baseline (p<0.05) compared to the SRP groups. These differences persisted throughout the 6-month period (p<0.05). There was also a reduction in mean CAL after all treatments (p<0.05) with no significant differences between the treatments (p>0.05). Additionally, a reduction in BOP was observed from baseline to 6 months (p<0.05) with no significant differences between treatments (p>0.05). A similar reduction (up to 6 months) was noted for PI (p<0.05). Furthermore, the patients with diabetes consistently presented a greater plaque score (p<0.05) when compared to control patients across all periods.

**Table 1:** Periodontal clinical parameters for each group and period. (n=38 patients; SD – standard deviation).

		Baseline	1 month	3 months	6 months
	**Groups n=38**	**Mean ± SD**	**Mean ± SD**	**Mean ± SD**	**Mean ± SD**
Probing depth (mm)	DSRP	2.71 ± 0.35^A.b^	2.38 ± 0.24^A.a^	2.42 ± 0.32^A.a^	2.38 ± 0.42^A.a^
	DPDT	3.03 ± 0.73^B.b^	2.72 ± 0.81^B.a^	2.65 ± 0.65^B.a^	2.78 ± 0.61^B.a^
	CSRP	2.77 ± 0.27^A.b^	2.4 ± 0.21^A.a^	2.32 ± 0.27^A.a^	2.36 ± 0.39^A.a^
	CPDT	3.29 ± 0.54^B.b^	2.81 ± 0.41^B.a^	2.78 ± 0.46^B.a^	2.65 ± 0.39^B.a^
Clinical attachment level (mm)	DSRP	2.39 ± 2.90^b^	2.04 ± 2.80^a^	2.09 ± 2.47^a^	2.13 ± 2.75^a^
	DPDT	2.36 ± 1.13^b^	2.14 ± 1.16^a^	2.15 ± 1.23^a^	2.2 ± 1.15^a^
	CSRP	1.88 ± 1.09^b^	1.72 ± 1.29^a^	1.59 ± 1.14^a^	1.49 ± 1.06^a^
	CPDT	2.64 ± 0.87^b^	2.14 ± 0.70^a^	2.15 ± 0.72^a^	2.08 ± 1.04^a^
Bleeding on probing (%)	DSRP	45.99 ± 13.41^b^	24.09 ± 9.22^a^	30.59 ± 15.53^a^	28.00 ± 13.68^a^
	DPDT	42.00 ± 23.01^b^	30.02 ± 16.87^a^	24.92 ± 9.79^a^	29.39 ± 12.16^a^
	CSRP	45.13 ± 21.62^b^	25.38 ± 27.71^a^	16.36 ± 10.14^a^	23.90 ± 16.53^a^
	CPDT	50.55 ± 13.58^b^	27.38 ± 8.92^a^	28.35 ± 17.31^a^	27.67 ± 15.16^a^
Plaque index (%)	DSRP	88.75 ± 10.95^B.b^	64.50 ± 25.11^B.a^	49.87 ± 24.20^B.a^	57.69 ± 29.30^B.a^
	DPDT	79.88 ± 20.04^B.b^	46.55 ± 18.39^B.a^	47.52 ± 23.96^B.a^	46.13 ± 21.02^B.a^
	CSRP	76.16 ± 17.50^A.b^	29.97 ± 15.46^A.a^	27.56 ± 16.10^A.a^	24.61 ± 13.30^A.a^
	CPDT	70.57 25.16^A.b^	39.36 ± 14.59^A.a^	38.37 ± 22.03^A.a^	31.09 ± 13.60^A.a^

Lowercase different letters = p<0.05 between time periods. Uppercase different letters = p<0.05 between groups. SRP – scaling and root planning. aPDT – antimicrobial photodynamic therapy. D – Type 1 diabetes mellitus patients. C – normoglycemic control patients.

**Figure 2:**
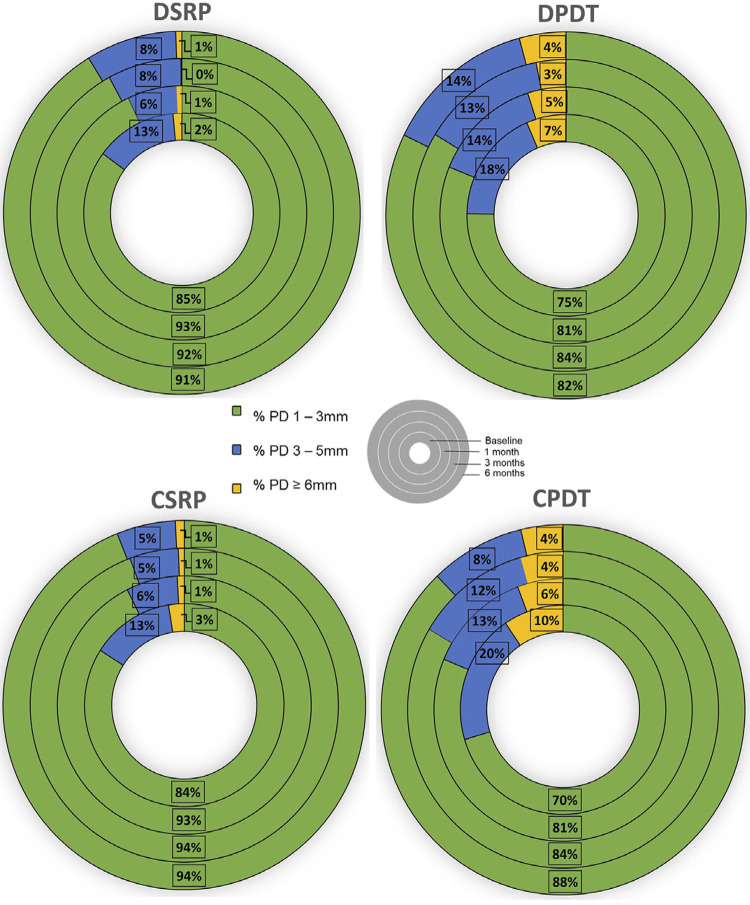
Percentage of healthy/shallow, moderate and deep pockets (by probing depths) at baseline, 1 month, 3 months and 6 months. Note the reduction in percentage of moderate and deep pockets and the increase in healthy sites from baseline to 6 months (p<0.05) for both groups (p>0.05).

Figure 2 illustrates PD for each group, categorized by the percentage of healthy/shallow, moderate and deep pockets. A reduction in moderate and deep periodontal pockets and an increase in healthy sites were observed from baseline to 6 months for both groups (p<0.05).

**Figure 3:**
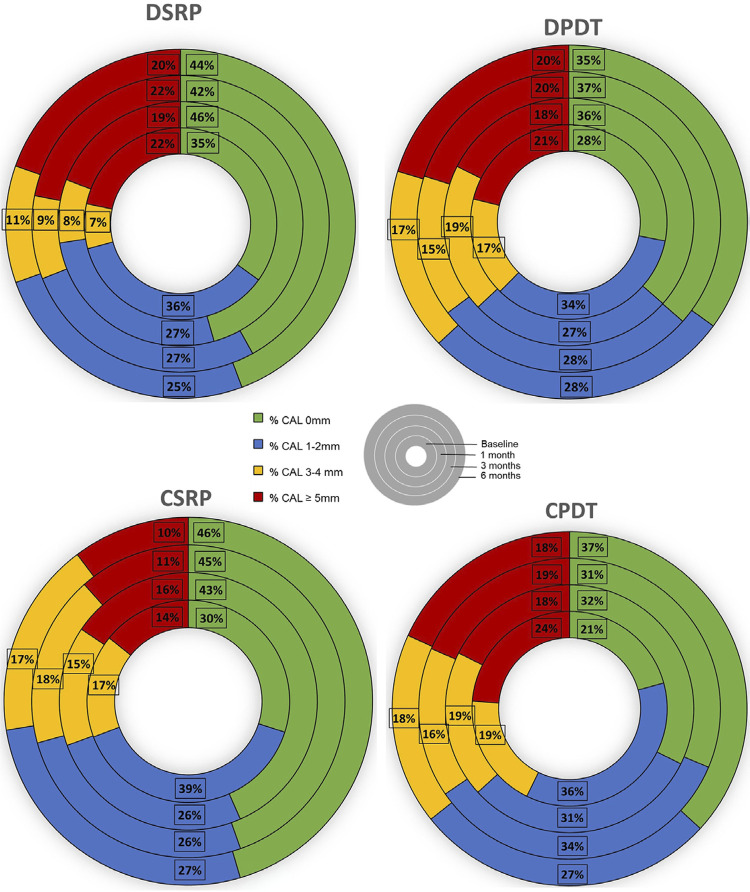
Percentage of sites with no loss of attachment, as well as initial, moderate, and severe loss of attachment (by clinical attachment level measurement) at baseline, 1 month, 3 months and 6 months. Note the reduction in percentage of initial and severe attachment loss and the increase in sites with no attachment loss from baseline to 6 months (p<0.05) for both groups (p>0.05). The percentage of sites with moderate attachment loss was similar for all periods and for both groups (p>0.05).

Figure 3 illustrates CAL for each group divided into four categories: no attachment loss, as well as initial, moderate, and severe attachment loss. There was a significant reduction in initial and severe attachment loss, coupled with an increase in sites without attachment loss (p<0.05). Notably, moderate attachment loss maintained its values over time for all groups. ( >0.05).

Differences in PD and CAL comparing each period with baseline are presented in Table 2. All groups exhibited a decrease in PD at one month; this reduction was sustained for 6 months (p>0.05), except for the CPDT group, which displayed a progressively greater reduction in PD until 6 months (p<0.05). Among T1D patients treated with aPDT, there was a significant reduction in PD at 3 months (0.38 mm - p<0.05), while patients in the control group showed the most substantial reduction at 6 months (0.66 mm – p<0.05). Normoglycemic patients exhibited a greater reduction in PD compared to T1D patients after 6 months of aPDT treatment (p<0.05). The most significant decrease in CAL levels was observed at one month, and this improvement remained stable for 6 months in control patients treated with aPDT (p<0.05).

**Table 2:** Reduction of probing depth (PD) and clinical attachment level (CAL) in time-interval, per site (n= 5441 sites). Negative numbers indicate reduction in PD and CAL. (SD - standard deviation)

GROUP		Period 1month -baseline	Period 3 months - baseline	Period 6 months - baseline
	**n (sites)**	**Mean PD ± SD (mm)**	**Mean PD ± SD (mm)**	**Mean PD ± SD (mm)**
DSRP	1122	-0.33 ± 0.82^a.b.c^	-0.25 ± 0.81^g^	-0.36 ± 0.85^a.b.c^
DPDT	1524	-0.32 ± 0.94^a.c.g^	-0.38 ± 0.97^a.b.e.f^	-0.25 ± 0.99^c.g^
CSRP	1409	-0.36 ± 0.73^a.c.f.g^	-0.45 ± 0.81^b.d.e^	-0.41 ± 0.86^a.b.d.e.f^
CPDT	1386	-0.48 ± 1.06^d.e.f^	-0.51 ± 1.18^d^	-0.66 ± 1.17^h^
Total	5441	-0.38 ± 0.90	-0.41 ± 0.96	-0.42 ± 0.99
	**n (sites)**	**Mean CAL ± SD (mm)**	**Mean CAL ± SD (mm)**	**Mean CAL ± SD (mm)**
DSRP	1122	-0.31 ± 1.37^a.b.d.e^	-0.12 ± 1.36^c^	-0.22 ± 1.35^a.b.c.e^
DPDT	1524	-0.23 ± 1.66^a.b.c.e^	-0.21 ± 1.62^a.b.c^	-0.17 ± 1.70^a.b.c^
CSRP	1409	-0.20 ± 1.12^b.c^	-0.34 ± 1.17^a.d.e^	-0.40 ± 1.16^d.e.f^
CPDT	1386	-0.48 ± 1.58^d.f^	-0.48 ± 1.71^d.f^	-0.58 ± 1.69^f^
Total	5441	-0.31 ± 1.46	-0.29 ± 1.50	-0.34 ± 1.51

Different lowercase letters = statistically significant differences among periods and groups (p<0.05). SRP – scaling and root planning. aPDT – antimicrobial photodynamic therapy. D – Type 1 diabetes mellitus patients. C – normoglycemic control patients.

Most patients successfully achieved the clinical endpoint for treatment (≤4 sites with PD≥ mm) after 6 months^23^ (Table 3). There were no significant differences in relation to systemic condition or treatment after 6 months (p>0.05). Both type 1 diabetes patients (65%) and normoglycemic patients (72%) achieved the clinical endpoint regardless of whether they received aPDT (60%) or SRP (88%) as their treatment (p>0.05).

**Table 3:** Achievement of clinical endpoint treatment (≤4 sites with probing depth ≥5mm) after 6 months of treatment. (SRP -scaling and root planing; aPDT – antimicrobial photodynamic therapy).

Systemic condition							
Achieved clinical endpoind Normoglycemic			Diabetes			Chi-square test	
Yes	13 (65%)		13 (72.2%)			p = 0.898	
No	7 (35%)		5 (27.7%)				
Total	20 (100%)		18 (100%)				
**Treatment**							
**Achieved clinical endpoind aPDT**			**SRP**			**Fisher p value**	
Yes	12 (60%)		16 (88.88%)			p= 0.067	
No	8 (40%)		2 (11.11%)				
Total	20 (100%)		18 (100%)				
**Systemic condition Treatment**		**Yes**	**% Yes**	**No**	**% No**		
Diabetes aPDT		6	60	4	40	Chi-square test	
Diabetes SRP		7	87.5	1	12.5	p =0.053	
Normoglycemic aPDT		4	40	6	60		
Normoglycemic SRP		9	90	1	10		
Total		26		12			

### Inflammatory cytokines

Quantification of cytokines revealed no differences in IL-1, IL -4, IL-6, IL-8 levels across different periods or treatments (p > 0.05). However, lower levels of TNF-α were observed in groups treated with aPDT (total value 1.48 pg/ml/ng) when compared to SRP groups (total value 2.65 pg/ml/ng) (p<0.05). (Table 4)

**Table 4:** Cytokine levels (pg/ml/ng) collected in 3 sites (n=72)/patient (n=24). (SD= Standard Deviation).

Systemic Condition	Treatment	IL-1 (Baseline)		IL-1 (3 months)		IL-1 (6 months)	
		(pg/ml/ng)		(pg/ml/ng)		(pg/ml/ng)	
		Mean	SD	Mean	SD	Mean	SD
Diabetes	aPDT	28.79	25.12	16.38	14.99	19.97	20.86
Diabetes	SRP	19.64	20.26	10.38	7.98	8.20	8.01
Control	SRP	17.50	25.27	15.03	36.15	23.80	71.35
Control	aPDT	33.00	36.79	16.44	18.29	26.29	43.92
**Systemic Condition**	**Treatment**	**IL-4 (Baseline)**		**IL-4 (3 months)**		**IL-4 (6 months)**	
		**Mean**	**SD**	**Mean**	**SD**	**Mean**	**SD**
Diabetes	aPDT	2.41	1.76	4.70	3.54	4.13	2.15
Diabetes	SRP	2.80	2.68	3.32	1.27	3.48	1.88
Control	SRP	5.36	7.44	5.04	3.55	3.73	1.77
Control	aPDT	2.82	1.32	3.92	2.37	3.53	1.56
**Systemic Condition**	**Treatment**	**IL-6 (Baseline)**		**IL-6 (3 months)**		**IL-6 (6 months)**	
		**Mean**	**SD**	**Mean**	**SD**	**Mean**	**SD**
Diabetes	aPDT	0.85	0.82	1.17	1.18	1.46	1.56
Diabetes	SRP	4.02	9.00	1.50	1.34	2.85	4.58
Control	SRP	2.21	6.68	0.83	0.77	1.71	2.99
Control	aPDT	1.22	2.25	1.76	2.01	0.96	1.10
**Systemic Condition**	**Treatment**	**IL-8 (Baseline)**		**IL-8 (3 months)**		**IL-8 (6 months)**	
		**Mean**	**SD**	**Mean**	**SD**	**Mean**	**SD**
Diabetes	aPDT	59.59	68.53	170.54	202.11	103.94	72.92
Diabetes	SRP	61.67	92.73	66.21	47.04	64.72	32.83
Control	SRP	117.66	245.92	90.95	89.65	88.26	66.20
Control	aPDT	87.30	59.85	98.93	64.02	101.76	83.69
**Systemic Condition**	Treatment *p<0.05	**TNF-alfa (Baseline)**		**TNF-alfa (3 months)**		**TNF-alfa (6 months)**	
		**Mean**	**SD**	**Mean**	**SD**	**Mean**	**SD**
Diabetes	aPDT	1.34	1.15	2.00	1.30	1.13	0.58
Diabetes	SRP	4.59	7.76	3.46	4.09	2.72	2.80
Control	SRP	1.85	1.83	2.26	1.78	1.53	1.23
Control	aPDT	1.06	0.74	1.66	1.26	1.73	3.06

SRP – scaling and root planning. aPDT – antimicrobial photodynamic therapy. D – Type 1 diabetes mellitus patients; C – normoglycemic control patients * = p<0.05 between treatments.

Among the T1D groups, a greater number of significant correlations were observed between probing depths and cytokines (Table 5). A negative correlation was found between PD and IL-4, indicating that as PD decreases, IL-4 levels increase. Additionally, strong positive correlations between PD and IL-1 were observed in the aPDT groups (p<0.05). This suggests that a greater reduction in PD is associated with lower levels of IL-1, a pro-inflammatory cytokine linked to periodontal inflammation.

**Table 5:** Pearson’s correlations (r values – p<0.05) between probing depth and cytokines.

	Diabetes - aPDT	Diabetes - SRP	Control - aPDT	Control - SRP
PD 3 months	IL-1 (0.6510)	x	x	x
	IL-4 (-0.486)			
PD 6 months	IL-1 (0.761)		IL-1 (0.625)	x
	IL-4 (-0.672)	IL-4 (-0.684)	IL-4 (-0.490)	

## Discussion

To the best of our knowledge, this is the first paper to attempt to describe the adjuvant effects of aPDT in SRP for treatment of periodontitis in patients with type 1 diabetes by means of periodontal clinical parameters and cytokine dosage. We also developed and described a novel statistical approach called site-by-site analysis. Treatment with aPDT reduced probing depths in T1D patients at 3 months and in normoglycemic patients (control group) up to 6 months. The most significant reduction in PD occurred at 6 months in the control group who underwent aPDT (p<0.05). Control patients exhibited greater reduction in CAL levels in both treatment groups (p<0.05). These findings suggest that normoglycemic patients respond more favorably to the treatment, while patients with T1D have a short-term response and should receive the therapy every three months.

In this study, IL-1beta, IL-4, IL-6, IL-8 and TNF-α were measured to confirm if adjuvant aPDT could reduce the local inflammatory burden in patients with diabetes and periodontitis. These cytokines were selected based on their previously reported association with periodontitis and poor glycemic control. IL-8 and IL-6 are expressed by human epithelial cells in response to *P. gingivalis* lipopolysaccharide in a high glucose environment.^25^ IL-1beta and IL-6 levels are elevated in gingival crevicular fluid of patients with periodontitis and poor glycemic control.^26^ In the aPDT groups, we observed significant correlations between the reduction of PD and the decrease in IL-1 (pro-inflammatory cytokine) as well as increase of IL-4, the latter of which is downregulated in periodontitis sites^27^ and serve as a pleiotropic anti-inflammatory cytokine that suppresses the pro-inflammatory environment, playing a protective role after periodontal treatment.^29^ Our results are in accordance with previous reports showing significant decrease of IL-1β and increase of IL-4 after non-surgical periodontal treatment.^28^ TNF-α is proinflammatory cytokine involved in bone resorption and the pathogenesis of periodontitis, and it is considered a marker of inflammatory disease.^29^ It has elevated values in both healthy and periodontitis sites in patients with diabetes.^24^ In this study, we observed lower levels of TNF-α on crevicular fluid from both groups treated with aPDT, suggesting a reduction in bone resorption. These findings are supported by studies that demonstrated a short-term reduction in inflammatory cytokines following aPDT treatment in normoglycemic patients.^30,31^ We suggest that aPDT treatment shifted the state from pro-inflammatory to anti-inflammatory in periodontal sites. We opted to conduct an immunological analysis rather than a microbiological one in this study due to its less frequent occurrence in the literature. The reduction of local cytokines is crucial for patients with chronic diseases such as diabetes, which often involve systemic inflammation. Local reduction of inflammation in the periodontium can be beneficial for these patients, assisting in the reduction of the overall inflammatory burden on their body.

In the current study, most patients reached the clinical endpoint (≤4 sites with PD≥5mm)^23^ within 6 months. No differences were observed among systemic conditions (T1D or normoglycemic) or treatments (SRP or aPDT). Similar results were observed in patients with T2D treated with different antibiotic protocols, with most of them reaching the clinical endpoint within 1 year using both protocols.^32^

The use of aPDT as an adjuvant periodontal treatment in normoglycemic patients remains controversial. Certain studies have suggested a significant short-term effect of this therapy on reducing PD and CAL.^33,34^ Our findings align with these results, demonstrating improvements in periodontal parameters at 3 and 6 months for both normoglycemic and in T1D patients. However, other studies have not reported statistically significant results in favor of aPDT.^35^ This controversy arises from differences in periodontitis diagnosis and patient risk profile, as well as variations in laser and photosensitizer protocols, standardization of experimental groups and the design of clinical trials.^36,37^

The frequency of therapy application is another crucial aspect of aPDT.^20^ In this study, we opted for multiple sessions based on previous research that demonstrated additional benefits in periodontal health for normoglycemic patients,^8-10,19^reductions in periodontal clinical parameters for T1D patients,^12^ and decreased levels of periodontopathogenic bacteria following multiple sessions of aPDT.^20^

Regarding the photosensitizer, this study used methylene blue at a concentration similar to that of Helbo^®^ blue sensitizer (10mg/ml) (Bredent medical GmbH & Co KG – Germany). Clinical studies employing this concentration of photosensitizer have demonstrated improvement of clinical periodontal parameters,^10,38-39^as well as reductions in periodontopathogenic bacteria^40-42^ and successful treatment of aggressive periodontitis.^43^ Concerns on the use of high concentrations of photosensitizer may emerge, as it could lead to optical shielding and increased light reflection thereby impeding photochemical reactions. We would like to propose that the positive clinical effects observed in the aforementioned studies, as well as in ours, can be attributed to two factors. First is the bactericidal effect of photosensitizer; a prior study by our research group demonstrated that blue photosensitizers at high concentrations (10 mg/ml) are capable of eliminating *Aggregatibacter actinomycetemcomitans in vitro* without additional light sources.^6^ Another factor is the dilution of photosensitizers in instrumented periodontal pockets, as in healthy periodontal sites, the volume of gingival fluid is approximately 0.27µl each 30s and in periodontal pockets it increases up to 0.88 µl each 30s.^44^ In aPDT, the photosensitizer is applied after scaling and root planing, which induces an increased inflammatory exudate and bleeding, leading to a greater dilution of photosensitizer. Furthermore, the use of a fiber optic periodontal probe with continuous movement from the bottom to the top of the periodontal pocket ensures that the light interacts with the entire volume of the photosensitizer, potentially mitigating the shield effect. This discussion could serve as focal point for future research.

A limitation of the present study was the method of analysis for the results of an incomplete sample (ideal n =13 patients/ group). We were able to overcome this issue by creating a site-by-site analysis, a novel way to analyze the effects of therapy on each periodontal site. In this way, we increased the sample size by a factor of 100 (ideal n = 52, study n = 38 and sites n= 5,441). This increase in the sample size through site-by-site analysis was able to reveal the different effects of both therapies (Table 1 compared to Table 2 and 3). Statistically significant differences typically do not emerge in studies that analyze solely the mean values of clinical parameters.^11-13^ We suggest the use of our site-by-site analysis in further clinical trials, as it is a more accurate way to individually evaluate the effects of treatment in each periodontal site. Another limitation of our study was the standardization of treatment groups (SRP or aPDT) by similar mean PD at baseline (p>0.05). Although all groups were similar in relation to CAL, both aPDT groups (T1D and normoglycemic) presented greater probing depths than SRP groups at baseline (Table 1). To overcome this problem, we analyzed the reduction of PD in a time-interval (1 month - baseline/ 3 months – baseline/ 6 months – baseline). In this way the absolute reduction in PD was analyzed and a favorable result for aPDT was evidenced (Table 2 and 3). A recent meta-analysis demonstrated that combining SRP with adjunctive aPDT effectively reduced HbA1c levels in the short term, establishing it as an important treatment for glycemic control in patients with type 2 diabetes.^45^ HbA1c is as a valuable marker for evaluating patients with T1D undergoing aPDT. HbA1c measurements represented a limitation in the current study. We were unable to compare the baseline HbA1C levels within 6 months, as patients had the habit of doing the laboratory exam only once a year in a public health service.

Despite the limitations of the present study, its contribution to the literature and scientific evidence by studying a population that is not the target of most studies (patients with T1D) should be highlighted. Our novel statistical evaluation method called site-by-site analysis used a recent proposal of achievement of a clinical endpoint^23^ to analyze the clinical effectiveness of therapies, and it showed that aPDT can reduce the inflammatory burden in periodontal tissues.

The time and cost for aPDT are also important factors; a drawback of this therapy is its increase in treatment time. Considering 1 minute of pre-irradiation and 80 to 90s of laser irradiation for each tooth, plus the time required for sulcus/pocket irrigation, a total of 40 to 60 minutes of aPDT is necessary for a patient presenting all teeth. In terms of costs, a low-level diode laser device can be seen as a savvy investment, as it plays a dual role in both aPDT and photobiomodulation therapy. Furthermore, the methylene blue photosensitizer is very cost-effective, thus leading to a positive cost-benefit ratio.

Considering the limited number of controlled studies and its controversial results, more randomized clinical trials with long-term follow-ups and diverse patient profiles ought to be conducted to establish robust scientific evidence regarding the benefits of aPDT in periodontal therapy. Additionally, more randomized clinical trials on adjuvant periodontal treatments for patients with T1D will further contribute to building solid scientific evidence for understanding its advantages within this specific population.

## Conclusion

Adjuvant aPDT on non-surgical periodontal treatment reduced periodontal clinical parameters and inflammatory cytokines in both patients with type 1 diabetes mellitus and normoglycemic patients. Normoglycemic patients with periodontitis exhibited a more favorable response to adjuvant aPDT treatment.
